# Identification and Characterization of the Phosphate-Solubilizing Bacterium *Pantoea* sp. S32 in Reclamation Soil in Shanxi, China

**DOI:** 10.3389/fmicb.2019.02171

**Published:** 2019-09-19

**Authors:** Qian Chen, Shanjiang Liu

**Affiliations:** Institute of Plant Nutrition and Resources, Beijing Academy of Agriculture and Forestry Sciences, Beijing, China

**Keywords:** PSB, *Pantoea* sp., phosphatase activity, reclaimed soil, pot experiment

## Abstract

Phosphate solubilizing bacteria (PSB) can convert insoluble forms of phosphorus (P) to accessible forms. Five highly efficient PSB strains, H22, Y11, Y14, Y34, and S32, were screened and isolated from an alfalfa rhizosphere in heavy metal-contaminated reclamation area in Shanxi Province, China. Based on morphological observations, 16S rRNA sequencing, cellular fatty acid composition analysis, and the BIOLOG test, H22, Y11, and Y34 were identified as *Pseudomonas* sp., while Y14 and S32 were identified as *Pantoea* sp. Among them, S32 showed the highest P-solubilizing efficiency in culture medium containing Ca_3_(PO_4_)_2_, lecithin, and powered phosphate rock. The culture medium conditions to obtain the highest P-solubilization efficiency were optimized as follows: the culture temperature was 30°C; the incubation time was 5 days; the initial pH was 7.0; and glucose served as the carbon source. Furthermore, the P-solubilization efficiency of S32 in media containing CaHPO_4_, lecithin, phosphate rock (PR), FePO_4_, or AlPO_4_ was determined to be 18.38, 3.07, 0.16, 0.51, or 2.62%, respectively. In addition, the acid and alkali phosphatase activities of S32 were tested as 6.94 U/100 mL and 4.12 U/100 mL, respectively. The soil inoculation experiment indicated that inoculation with S32 resulted in an obvious improvement in the available P of both the experimental and reclaimed soil. The rice seedling growth experiment also suggested that the application of S32 significantly increased the plant height, biomass, root growth, and P uptake of rice in both experimental and reclaimed soil. Taken together, the isolated S32 strain showed high P-solubilization capacity for both Pi and Po, and its ameliorative effect on reclaimed soil recovery provides the theoretical basis for crop development in the reclaimed soil of mine field.

## Introduction

Phosphorus (P) is the second most important nutrient for plant growth, accounting for 0.2% (w/w) of plant dry weight (Maharajan et al., 2018). P plays an irreplaceable role in the ecosystem by participating in most aspects of energy metabolism, nucleic acid and protein synthesis, and kinase regulation (Nesme et al., 2018). The average P content in soil is nearly 0.05% (w/w) with the main two forms being inorganic P (Pi) and organic P (Po). Nevertheless, only 0.1% of P can be utilized by plants, rendering available P a restrictive factor for plant growth (Lambers and Plaxton, 2018). Phosphate anions in chemical fertilizer available to plants are extremely reactive and become fixed through interactions with Ca^2+^, Fe^3+^, and Al^3+^ ions in the soil to form insoluble phosphate salt complexes; however, the plant utilization efficiency for P in chemical fertilizers is only 5–25% (Schnug and Haneklaus, 2016), leading to P enrichment in the soil and the loss of soil fertility. These realities make increasing the P utilization rate for plant growth an urgently needing situation.

Phosphate solubilizing bacteria (PSBs) convert unavailable P (both Pi and Po) into available P to satisfy the requirements of plants through dissolution and absorption. According to the various P-dissolving patterns, PSBs can be divided into two classes: (1) Pi-solubilizing microorganisms that secrete organic acid to dissolve Pi compounds and (2) Po-mineralizing microorganisms that secrete phosphatase to enzymatically mineralize Po compounds. The application of both classes of PSBs in soil decreases the pH of the soil and forms a P-offering microarea around the plant rhizosphere, consequently improving the P supply available to the plant and strengthening the activity of other beneficial microorganisms, such as *Rhizobium* and *Trichoderma*. These applications promote the absorption of nutritive element ions.

Recently, following the development of energy bases and mines explored in Shanxi Province, China, a vast amount of cultivated land was contaminated with gangue, mineral waste, heavy metal, and rock debris. Approximately 68 km^2^ of cultivated land lost their crop productivity. Therefore, rational and scientific measures for soil reclamation in the mining area should be developed and applied without delay. In this study, to acquire a high PSB strain and improve the soluble P available to plants in the reclaimed soil, five PSBs were screened from the alfalfa rhizosphere soil of the reclamation area in the mining area of Xiao Yi town in Shanxi Province, China. Their P-solubilizing capacities under different conditions were investigated. The effect of PSB S32 on P solubility in the reclaimed soil recovery, plant and root growth, and the P uptake of rice were also evaluated. The present data suggest that the application of the isolated PSB would be of great importance in the bioremediation of reclaimed soil in mining areas.

## Materials and Methods

### Reclaimed Soil

Approximately 200 g of soil samples with three replicates were taken from the rhizosphere of alfalfa in reclaimed soil of the Xiao Yi opencast mine area (37.15N; 111.78E) in Shanxi Province in July 2013. For each sample, the roots of vigorously growing plants were carefully dug. The bulk soils loosely bound on the roots were shaken off and discarded. The soil still attached to the plant roots was swept with a brush and collected as the rhizosphere soil sample (Zhu et al., 2017). The sealed soil samples were immediately placed in a refrigerated container. Each sample was used to isolate PSB immediately after bringing it back to the laboratory. The remaining part of the sample was then homogenized, air dried, and passed through a standard 100 sieve to analyze its physicochemical properties and P fractions. Triplicate samples were used for all experiments.

### Physical and Chemical Properties of Reclaimed Soil

The reclaimed soil was aseptically separated from the roots to assess the physical and chemical properties. The pH was measured in a 2:1 water:soil suspension with a pH meter (Corey, 1971). Soluble P was extracted by the bicarbonate method and was then analyzed by the Molybdenum blue method (Colwell, 1963). The organic matter content was measured using the potassium dichromate colorimetric method (Nelson et al., 1996). The available potassium content was determined using the flame photometer method (Gammon, 1951). The content of nitrate (NO_3_-N) and ammonium nitrogen (NH_4_^+^-N) extracted by a potassium chloride solution was determined with an AutoAnalyzer 3 (AA3) equipped with NH_4_^+^ and NO_3_ channels (SEAL, Germany) (Crooke and Simpson, 2010). Briefly, 1–2 g of soil that had passed a 2-mm sieve were placed into a 50 mL Erlenmeyer flask. Twenty mL of KCl solution was added to the flask. Samples were shaken on a horizontal shaker for 1 h and filtered through Whatman No. 2 filter paper. Filtrates were analyzed for NH_4_^+^-N, and NO_3_-N. Extractions of all soils were repeated three times. Data are presented as the means ± standard deviation. The physicochemical properties of the soil samples are shown in Table 1.

**TABLE 1:** Physicochemical characteristics and nutrients in reclamation and experimental soil.

**Sites**	**pH**	**M.C**	**O.M**	**Nitrate N**	**Ammonium N**	**Available P**	**Available K**	**Total N**	**Total P**	**Total K**
		**(g/kg)**	**(g/kg)**	**(mg/kg)**	**(mg/kg)**	**(mg/kg)**	**(mg/kg)**	**(mg/kg)**	**(mg/kg)**	**(mg/kg)**
4-year Reclamation soil	6.21	15.2	7.00	5.92	19.20	4.11	150.36	395.25	361.51	4625.84
Experimental soil	4.0	22.3	26.54	8.12	29.35	6.39	355.90	676.22	480.36	5160.02

*MC, moisture content, OM, organic matter.*

### Culture Medium

Pi culture medium (Sahu and Jana, 2000) consisted of 10.0 g glucose, 0.5 g (NH_4_)_2_SO_4_, 0.5 g yeast extract powder, 0.3 g NaCl, 0.3 g KCl, 0.03 g FeSO_4_ ⋅ 7H_2_O, 0.3 g MgSO_4_ ⋅ 7H_2_O, 0.03 g MnSO_4_ ⋅ 4H_2_O, 5.0 g Ca_3_(PO_4_)_2_, 1000 mL distilled water, and 20.0 g agar, pH 7.0–7.5.

Po culture medium (Surange et al., 1997) consisted of 10.0 g glucose, 0.5 g (NH_4_)_2_SO_4_, 0.5 g yeast extract powder, 0.3 g NaCl, 0.3 g KCl, 0.03 g FeSO_4_ ⋅ 7H_2_O, 0.3 g MgSO_4_ ⋅ 7H_2_O, 0.03 g MnSO_4_ ⋅ 4H_2_O, 1.0 g CaCO_3_, 0.2 g lecithin, 1000 mL distilled water, and 20.0 g agar, pH 7.0–7.5.

Nutrient agar culture medium (Deshmukh, 2007) consisted of 10.0 g peptone, 3.0 g beef extract, 5.0 g NaCl, 1,000 mL distilled water, and 15–20 g agar, pH 7.2–7.4.

Tryptic soy broth (TSB) culture medium (Mishra and Goel, 1999) contained 15.0 g tryptone, 5.0 g soy peptone, 5.0 g NaCl, and 1000 mL distilled water, pH 7.2–7.4.

BUG (BIOLOG Universal Growth agar) culture medium contained 57.0 g BUG agar culture (BLG.70101, BIOLOG) and 1000 mL distilled water, pH 7.0–7.5.

### Positive Control Bacteria

The positive control bacterium *Bacillus megaterium As*1.223, which serves as a P-solubilizing microbial fertilizer in common use (RodrìGuez and Fraga, 1999; Chen et al., 2006), was supplied by the Microbe Collection Center of the Chinese Academy of Sciences (CAS).

### Isolation and Determination of the P-Solubilization Ability of Bacteria

The bacterial isolation protocol and determination of the P-solubilization ability were performed as previously described (Chen et al., 2014). Briefly, for isolation of bacteria, each soil sample were homogenized in sterile distilled water and serially diluted. Aliquots of each dilution were spread on Nutrient agar medium and incubated at 30°C for 24–48 h. Colonies were selected on the basis of the development of a clear halo; the clones were further purified on Nutrient agar and TSB media. Once purified, each isolate was stored at −80°C in the same medium with 20% (v/v) glycerol.

For P-solubilizing ability determination, the isolates were screened by culturing at 28°C on the media supplemented either lecithin (Po culture medium) or Ca_3_(PO_4_)_2_ (Pi culture medium). When the colonies appeared in 4–5 days, those causing a clear phosphate-solubilizing zone were selected out for further calculation. The size of phosphate-solubilizing zone was determined for each colony (Nair et al., 1995).

### Bacterial Colony and Mycelia Morphology Observation

Candidate bacteria were inoculated on agar media and cultured for 48 h at 30°C. The bacterial colony shape and color were observed, and bacteria were Gram stained. The morphology and size of the bacterial samples were observed by scanning electron microscopy (SEM) (S-3400N, Hitachi) at the Beijing Agricultural Biotechnology Center.

### Taxonomical Assignment

16S rRNA sequencing and phylogenetic analysis were performed for taxonomical assignment. The primers sequences are displaying as follows: 27 F: 5′-AGA GTT TGA TCC TGG CTC AG-3′, and 1492 R: 5′-TAC GGT TAC CTT GTT ACG ACT T-3′. Gene alignment was performed within the EzTaxon database, and a systematic phylogenetic analysis was performed using Mega 4.0 software. The phylogenetic tree was constructed by the neighbor-joining method with a bootstrap value of 1,000.

### Accession Numbers

The nucleotide sequence data reported in this paper appear with the following accession numbers (GenBank: MN294691, MN305765, MN305766, MN305767, and MN305768).

### Gas Chromatography (GC)-Fatty Acid Methyl Ester (FAMES) Analysis

Gas Chromatography-Fatty Acid Methyl Ester in each isolated PSB was determined using a Sherlock automatic bacterial identification system. All the identification procedures were completed by the Beijing Agricultural Biotechnology Research Center. TSB culture medium was applied for bacterial excitation. FAME profiles were then obtained by analyzing the samples on a GC (Agilent Technologies, United States) equipped with flame ionization detector (FID) and MIDI (R) Sherlock Microbial Identification System (MIDI Inc., Newark, DE, United States) software. FAMEs were identified according to their retention time, compared to a commercial standard mixture (MIS standard calibration, Part no. 1200-A) (Sasser, 1990).

### BIOLOG Identification

The BIOLOG microbial ID system (Biolog Inc., Hayward, CA, United States) was used to further identify the physiological fingerprint of isolated bacteria strains, in accordance with the manufacturer’s manual. Briefly, the strains were cultured in BUG media for 24 h generations at 30°C and resuspended in inoculating solution. Then, 100 μL of culture was inoculated into each well of a Gen III MicroPlate and incubated at 33°C in the dark for 4–6 h or 16–24 h. The bacterial metabolites of PSBs were obtained using a spectrophotometer and then analyzed and compared with the BIOLOG database.

### Determination of the P-Solubilization Ability

The S32 strain was cultured in Pi or Po media for 7 days with Ca_3_(PO_4_)_2_, lecithin, CaHPO_4_, powdered PR, FePO_4_, or AlPO_4_ as the P source. Non-inoculated medium was used as a blank control, while *B. megaterium As*1.223 was used as a positive control. The P-solubilization ability of the PSBs was tested three times independently. Each isolate was inoculated into a 200-mL conical flask containing 100 mL of Pi or Po liquid medium (as described above) and then shaken (160 rpm) at 30°. The suspensions were sampled at the indicated days postincubation. To remove the insoluble culture media, the suspensions were kept stationary for more than 1 h. The acidity was assayed simply by reading on a pH meter, and the P availability was determined with the Molybdenum blue method (Watanabe and Olsen, 1965). Optimum pH (4–10), temperature (20, 25, 30, 35, and 40°C), carbon source (Glu, Suc, Sta, Fru, Lac, Man, and Gly), and sodium content (1–10%, w/v) for P-solubilization in liquid media were determined following the above method.

### Alkaline and Acidic Phosphatase Activity Detection

Culture media supernatants from the lecithin-containing media were collected and detected using Alkaline and Acidic Phosphatase Kits (P0326 and P0321, Beyotime) in accordance with the manufacturer’s instructions. *B. megaterium As*1.223 and non-inoculated medium served as positive and negative controls, respectively. The phosphatase activity of 1 mg of phenol production after a 15 min reaction with the matrix at 30°C in 100 mL of medium was defined as one King-Armstrong unit.

### Soil Experiment

The S32 strain was cultured in 100 mL of TSB medium at 30°C for 36 h, and then cells were harvested by centrifugation at 5000 rpm at 4°C for 5 min and adjusted to 1 × 10^8^ cells/mL in sterile deionized water by dilution and plating. The experimental and reclaimed soil were autoclaved, and the initial available P was about 6.41 mg/kg and 4.05 mg/kg. For the experimental group, 600 g of soil was soaked with the above S32 strain suspensions (10^8^ cells/mL) for 30 min. Non-inoculated soil served as a negative control. The available P in the soil of each treatment group was determined at 10 and 30 days post administration, and the experiment was performed independently three times.

### Rice Seedling Growth Experiment

Sterilized deionized water-washed 7-day-old Malaysia MR-219 rice seedlings were grown in experimental and reclaimed soil. For the experimental group, homogenous seedlings were soaked with S32 strain suspensions (10^8^ cells/mL) for 30 min. Then, three seedlings were sown in each plastic pot. The plants were grown under natural growth conditions, maintaining a 20% absolute water content. Rice seedlings were harvested 21 days after sowing. Approximately 5 × 10^8^/mL of live washed bacterial S32 cells were used as inoculum in each bacterial treatment.

The root morphology of the MR-219 rice was determined using a root scanner (Expression 1680, Epson). Total root length (cm), total surface area (cm2) and total volume (cm3) were quantified using a scanner (Expression 1680, Epson) equipped with a 2 cm deep plexiglass tank (20.30 cm) filled with H_2_O (El Zemrany et al., 2007). The scanned data were processed by Win-Rhizo software (Regent Instruments Inc.).

### Organic Acid Evaluation

Approximately 20 mL of the samples from each treatment were injected into HPLC with a UV detector set at 210 nm. A Rezex ROA-organic acid “H^+^” (8%) column was used, and the mobile phase was 0.005 N H_2_SO_4_ with a flow rate of 0.17 mL/min.

### Statistical Analysis

All the data were processed with SPSS 13.0 statistical software and are presented as the mean ± standard deviation (SD). Student’s *t*-test analysis and one-way ANOVA was used to calculate the data variance, and *P* < 0.05 represents a significant difference.

## Results

### Screening and Isolation of the PSBs

**FIGURE 1:**
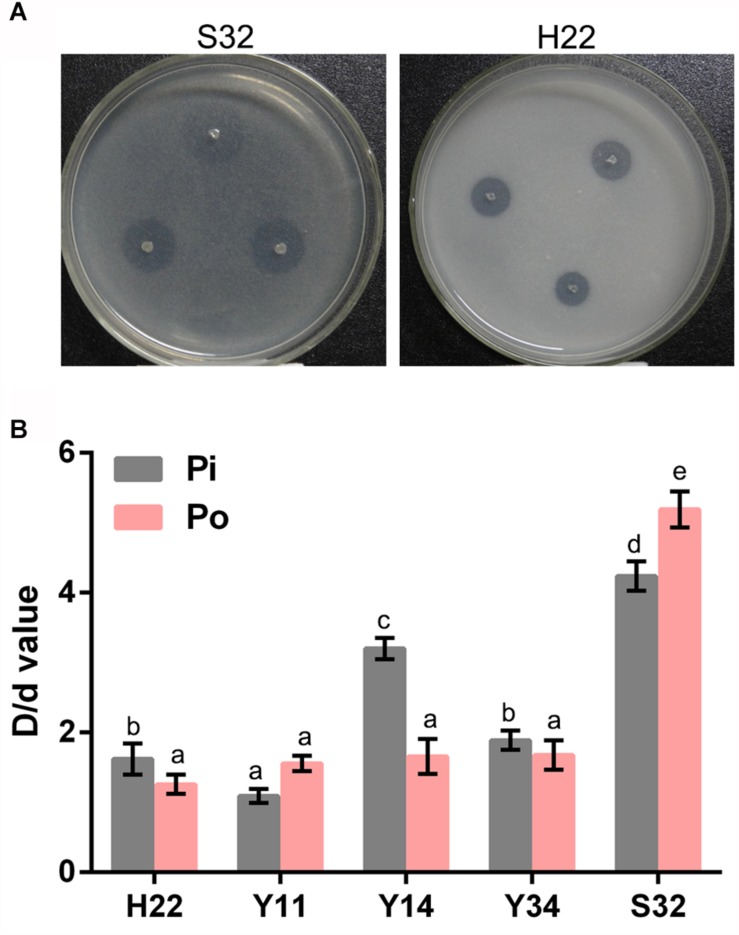
The bacterial colonies of PSBs. **(A)** The bacterial transparent halos of S32. **(B)** Comparison of the D/d value of each PSB. Different lowercase letters indicate significance at the 5% level.

A total of 20 bacteria with a considerable transparent zone were screened and isolated by colony formation on Pi and Po plating media (Figure 1A). Among them, there were five bacteria with a diameter of the transparent zone (D) to colony (d) ratio larger than 1.50, as formed on Pi plating media, and a ratio of D/d on Po plating media larger than 1.90. Isolation S32 displayed the highest D/d ratio on Pi medium and Po medium, 4.24 and 5.19, respectively, (Figure 1B).

**FIGURE 2:**
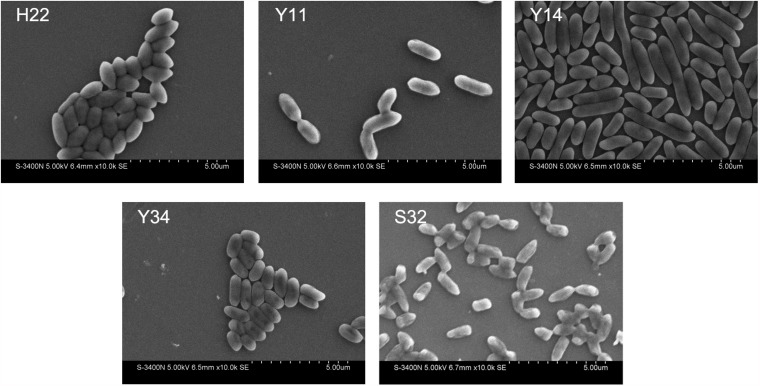
Morphology of each PSB by SEM (×10^4^).

Five PSBs (H22, Y11, Y14, Y34, and S32) formed semicylindrical colonies that were emulsus, yellow, opaque, glossy, and orderly. SEM revealed that the thallus was rhabditiform, non-spore forming, and gram-negative with a size of 0.5–0.6 μm × 0.6–1.6 μm (Figure 2).

### Characterization of PSBs

**FIGURE 3:**
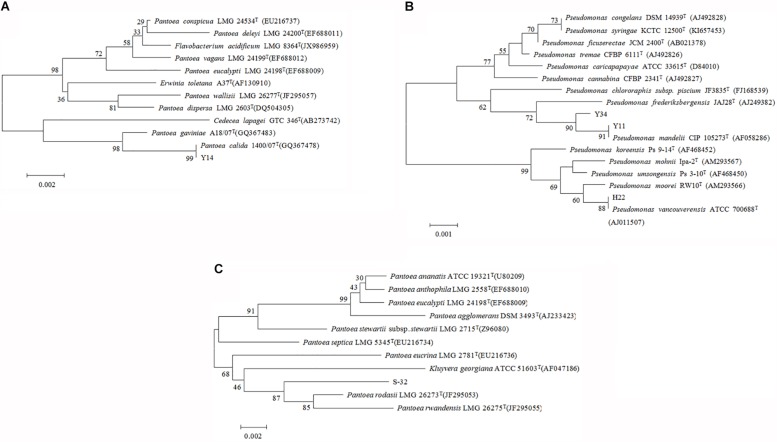
Phylogenetic tree of maximally similar species representing strains Y14 **(A)**, Y11, Y34, H22 **(B)**, S32 **(C)**.

The DNA gene size of H22, Y11, Y14, Y34, and S32 was approximately 1.5 kb. The alignment of the 16S RNA of five PSBs was performed with the EzTaxon database, and a phylogenetic analysis was performed with highly homologous strains as reference bacteria. We found that Y14 was homologous to *Pantoea calida* 1400/07^T^ (GQ367478) (Popp et al., 2010) (99.79%) (Figure 3A). H22, Y11, and Y34 were highly homologous to *Pseudomonas vancouverensis* ATCC 700688^T^ (AJ011507) (Mohn et al., 1999), *Pseudomonas mandelii* CIP 105273^T^ (AF058286) (Verhille et al., 1999), and *Pseudomonas frederiksbergensis* JAJ28^T^ (AJ249382) (Andersen et al., 2000), respectively, (Figure 3B). S32 was highly homologous to *Pantoea* sp., of which *Pantoea rodasii* LMG 26273^T^ (JF295053) (Brady et al., 2012) displayed the highest similarity (99.94%). S32 clustered with LMG 26273^T^ and LMG 26275^T^ in the phylogenetic tree (Figure 3C).

### Stress Resistance Test of Five PSBs

Each PSB displayed high tolerance to a broad range of temperatures, pH values, and sodium concentrations, especially for S32. Its tolerance ranged from 4 to 37°C, pH values of 4–11, and sodium concentrations of 2–10% (Supplementary Table S1).

### Fatty Acid Composition Analysis

Fatty acids were mainly distributed in the cell membrane as the basis of the lipids and lipopolysaccharides. The fatty acid composition data were analyzed according to the Microbial Identification System: the characteristic peak value >1% was C_12__:__0_, C_16__:__0_, C_17__:__0_cyclo, and C_18__:__1__ω__7c_ in the second and third peaks. The major characteristic peak contained C_12:0_ and C_18:1ω__7c_, and the third peak occupied 27.6, 14.6, and 25.3% of that of the whole-cell component, which was consistent with that of LMG 26273^T^ and LMG 26275^T^ (Supplementary Table S2).

### BIOLOG Identification

To further characterize the S32 strain, which showed the highest P-solubilizing capacity among all the isolated PSBs, a BIOLOG test was performed to identify its species. The alignment results of strain S32 displayed good agreement with *Pantoea cypripedii* at 22 h after culturing at 33°C (SIM value = 0.589). The BIOLOG reaction results ultimately confirmed that S32 was a *Pantoea* sp., which was consistent with our 16S rDNA sequence analysis (Supplementary Table S3).

### P-Solubilizing Efficiency of Five PSBs With Different P Sources

**FIGURE 4:**
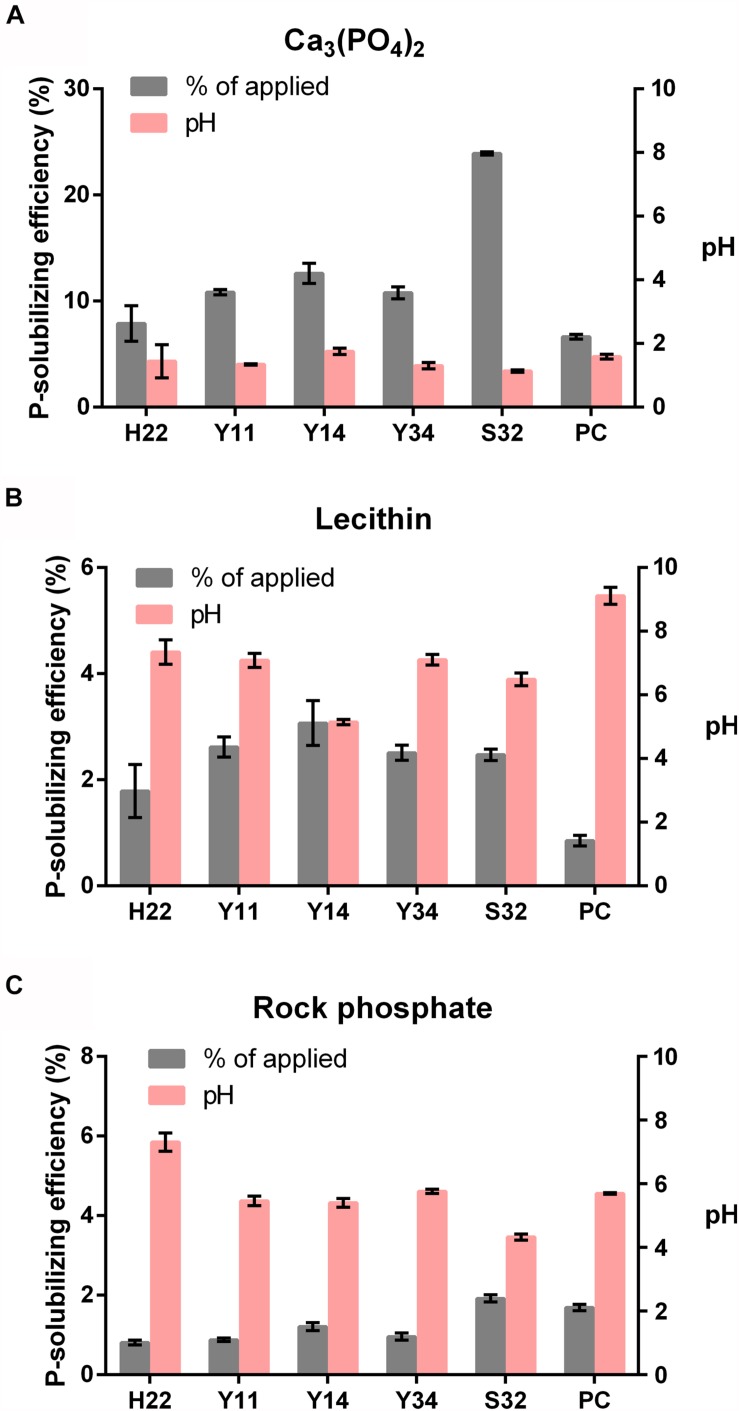
P-solubilization capability of PSBs with Ca_3_(PO_4_)_2_ **(A)**, lecithin **(B)**, and PR **(C)**.

To screen out the bacterium with the highest P-solubilizing efficiency in Pi, Po, and powdered PR, the five selected bacteria were inoculated into 100 mL of liquid media supplemented with either Ca_3_(PO_4_)_2_, lecithin, and powdered PR. For Ca_3_(PO_4_)_2_, the P-solubilizing efficiency of S32 reached 24.19%, which was nearly quadruple that of the positive control *B. megaterium As*1.223. The available P in the medium was 1256.67 mg/L, while the pH value was reduced by 3.51, which was double that of *As*1.223 (Figure 4A). The P-solubilizing efficiency for lecithin was 2.47%, which was 1.5 times that of the positive control *As*1.223. The available P content in the Po medium was 13.05 mg/L, and the pH value decreased by 3.89 (Figure 4B). However, with respect to PR, the five PSBs showed a degree of efficiency similar to that of *As*1.223 (Figure 4C).

### P-Solubilization Capability of S32 Under Different Experimental Conditions

**FIGURE 5:**
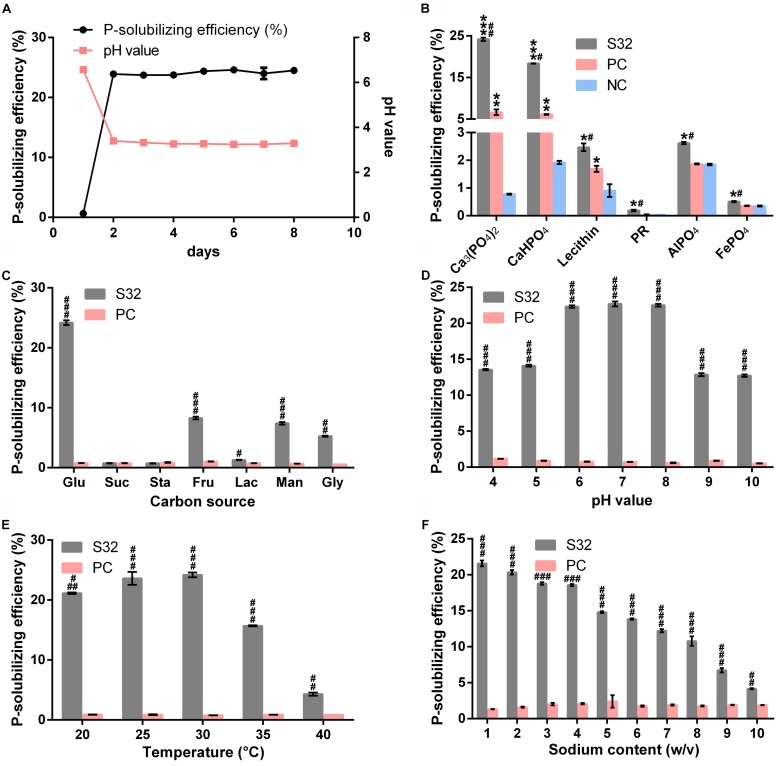
P decomposition rates of strain S32 for different incubation times **(A)**, P sources **(B)**, carbon sources **(C)**, pH values **(D)**, temperatures **(E)**, and sodium contents **(F)**. Data are shown as Mean ± SD. ^∗^*P* < 0.05, ^∗∗^*P* < 0.01, and *^∗∗∗^P* < 0.001 vs. NC group; ^#^*P* < 0.05, ^##^*P* < 0.01, and ^###^*P* < 0.001 vs. PC group.

To determine the effect of inoculation time on the P-solubilization rate of S32, media containing Ca_3_(PO_4_)_2_ was inoculated with S32 for different time points. The P-solubilization efficiency of S32 rapidly reached 23.91% at 24 h postinoculation (PI) and increased slowly to its maximum (24.61%) at day 5 PI. The content of available P in the medium reached 1204.67 mg/L at 24 h PI and then slowly peaked at 1332.33 mg/L at day 7 PI. The pH in the medium quickly decreased to 3.40 at 24 h PI, which was maintained for the following 6 days (Figure 5A).

The P-solubilizing efficiency of S32 on different P sources, Ca_3_(PO_4_)_2_, CaHPO_4_, lecithin, PR, AlPO_4_, and FePO_4__,_ was 24.09, 18.38, 2.47, 0.19, 0.51, and 2.62%, respectively. Each condition yielded a rate significantly higher than the *B. megaterium As*1.223 positive control. The pH value decreased by 3.51, 2.68, 3.89, 3.58, 3.46, and 4.03 in these types of media, respectively. Thus, the efficiency of P-solubilization on Ca_3_(PO_4_)_2_ was the greatest compared to the other P sources tested (Figure 5B). Notably, S32 showed significantly higher P-solubilizing efficiency than *B. megaterium As*1.223 positive control.

By using glucose, sucrose, starch, fructose, lactose, mannitol, or glycerin as the carbon source, the P solubilization efficiency of S32 was 24.19, 0.75, 0.73, 8.29, 1.30, 7.40, or 5.25%, respectively, (the pH of the medium was reduced by 3.51, 0.23, 0.51, 1.82, 0.73, 2.23, or 1.56, respectively). This result demonstrated that glucose was the best carbon source for assisting the P-dissolving capacity of S32. Notably, we found that sucrose and starch did not differ from the negative control, suggesting that these two saccharides cannot be utilized as carbon sources by S32 (Figure 5C).

To assess the influence of pH on the P solubilizing efficiency of S32, the initial pH value of the Pi medium was set as 4.0, 5.0, 6.0, 7.0, 8.0, 9.0, or 10.0. After culturing for 24 h, the P-dissolving rate of bacteria S32 in these media was 13.56, 14.10, 22.32, 22.69, 22.51, 12.87, or 12.72%, indicating that the highest P solubilizing rate occurred at pH = 7 (Figure 5D).

Next, we determined the effect of temperature on the P-dissolving rate of S32. The P-dissolving rate for Ca_3_(PO_4_)_2_ of bacteria S32 after it was cultured for 5 days at 20, 25, 30, 37, or 40°C was 21.13, 23.16, 24.19, 15.70, or 4.30% (the pH value of the medium was reduced by 3.15, 3.33, 3.51, 2.73, or 1.63), respectively. These data demonstrated that the best P-solubilizing efficiency was obtained at 30°C (Figure 5E).

The P-solubilizing ratio for Ca_3_(PO_4_)_2_ by S32 in growth medium containing NaCl with concentrations of 1, 2, 3, 4, 5, 6, 7, 8, 9, or 10% was 21.58, 20.35, 18.79, 18.57, 14.80, 13.85, 12.22, 10.79, 6.73, or 4.16% (the pH value in the medium was reduced by 2.90, 2.77, 2.58, 2.49, 2.70, 2.54, 2.37, 2.30, 1.87, or 1.13), respectively. These data suggested that the P-solubilizing capacity decreased with increasing sodium concentration (Figure 5F).

### Acid and Alkaline Phosphatase Activity of S32

The activity of acid phosphatase (ACP) in S32 was 6.94 U/100 mL, which was 3.6-fold greater than the positive control *As*1.223 (1.93 U/100 mL). The activity of alkaline phosphatase (ALP) in S32 was 4.12 U/100 mL, while that of the positive control *As*1.223 was 1.90 U/100 mL. These results clearly demonstrated that both ACP and ALP activity in S32 were higher than in *As*1.223.

### P-Dissolving Capacity in Experimental and Reclaimed Soil

The available P in the experimental soil of the S32 treatment group was increased by 8.85 mg/kg at 30 days post application, respectively. The difference between the before and after inoculation was significant (*P* < 0.05). Moreover, the available soil P of the S32 groups at 30 days post application displayed significant differences in many P fractions (Fe-bound P, Ca-bound P, moderately labile Po, and moderately resistant Po), suggesting that the S32 showed multiple P-solubilizing ability in experimental soil (Table 2). We also tested the P-solubilizing rate of S32 in Xiaoyi reclaimed soil which displayed obviously less Po than experimental soil, and the results showed that S32 also displayed a higher P-solubilizing efficiency in many P fractions (Occluded P, Ca-bound P, moderately labile Po, moderately resistant Po, and highly resistant Po) compared to data obtained before S32 inoculation (Table 3). We also found that S32 possessed higher P-solubilizing capacity than PC bacteria.

**TABLE 2:** Experimental soil culture test of strain S32.

**Groups**	**S32**		**PC**		**NC**	
			
**P fractions**	**Before**	**After**	**Before**	**After**	**Before**	**After**
Av-P	6.41	15.26	6.40	8.83	6.40	6.41
Al-P	1.66	1.04	1.55	1.58	1.64	1.64
Fe-P	13.44	12.58	13.31	12.64	13.36	13.32
Oc-P	82.50	82.14	82.25	82.48	81.84	81.82
Ca-P	113.42	105.00	112.57	112.56	116.80	116.48
L-Po	10.76	10.44	11.21	10.76	11.34	11.28
ML-Po	38.60	34.40	38.39	37.84	38.24	38.42
MR-Po	26.60	23.80	26.41	25.40	26.76	26.38
HR-Po	186.36	184.66	185.49	182.42	187.34	183.84

*Distributions of experimental soil P fractions before and after S32 inoculation. Av-P, available P; Al-P, Al-bound P; Fe-P, Fe-bound P; Oc-P, Occluded P; Ca-P, Ca-bound P; L-Po, labile organic P; ML-Po, moderately labile organic P; MR-Po, moderately resistant organic P; HR-Po, highly resistant organic P.*

**TABLE 3:** Experimental and reclaimed soil culture test of strain S32.

**Groups**	**S32**		**PC**		**NC**	
			
**P fractions**	**Before**	**After**	**Before**	**After**	**Before**	**After**
Av-P	4.05	12.89	4.02	6.90	4.00	4.05
Al-P	10.56	10.02	10.50	10.22	10.38	10.52
Fe-P	32.56	29.03	33.67	32.08	33.14	33.08
Oc-P	115.92	113.61	116.48	113.79	115.21	115.46
Ca-P	69.80	65.34	67.87	69.22	71.04	70.51
L-Po	9.00	8.36	8.83	9.36	9.03	8.76
ML-Po	19.54	17.02	18.60	16.31	19.00	19.05
MR-Po	14.22	11.02	14.45	13.34	14.30	14.24
HR-Po	51.60	46.44	50.62	49.88	51.22	51.06

*Distributions of reclaimed soil P fractions before and after S32 inoculation. Av-P, available P; Al-P, Al-bound P; Fe-P, Fe-bound P; Oc-P, Occluded P; Ca-P, Ca-bound P; L-Po, labile organic P; ML-Po, moderately labile organic P; MR-Po, moderately resistant organic P; HR-Po, highly resistant organic P.*

### The Effect of S32 on Plant Growth

We observed that plant height and dry biomass significantly increased following S32 inoculation. Higher plant height (14 cm) and heavier dry biomass (0.58 g) were observed in the S32 inoculated treatments (Table 4) compared with the positive control inoculation groups. In addition, we examined the morphology of the PSB-inoculated rice roots in both experimental and reclaimed soil. The root length, root surface area and volume varied with the different inoculations. Treatment with S32 improved rice root development, especially for the root surface area (Table 5). Generally, greater root length, surface area and volume were found in inoculated compared to positive control rice plants. In addition, we found a higher P concentration in both the leaves and roots of plants inoculated with S32, indicating that S32 increased the P uptake for plant growth (Table 6). The effect of PSB inoculation on the release of organic acids has also been determined through examination of the rhizospheric organic acid concentrations. The plants inoculated with S32 released the highest amounts of organic acids compared to the positive control group (Table 7).

**TABLE 4:** Effect of S32 on the height and biomass of plants in ES or RS.

**Treatment**	**Plant height**	**Dry weight**	**Plant height**	**Dry weight**
	**(cm) in ES**	**(g) in ES**	**(cm) in RS**	**(g) in RS**
S32	14.56 ± 0.27^∗^	0.58 ± 0.08	11.96 ± 0.20^∗^	0.39 ± 0.05^∗^
PC	12.27 ± 0.25	0.45 ± 0.17	9.20 ± 0.09	0.26 ± 0.10
NC	11.82 ± 0.35	0.42 ± 0.05	9.00 ± 0.11	0.24 ± 0.05

*ES, experimental soil; RS, reclaimed soil; PC, positive control; *B. megaterium As*1.223; NC, negative control. ^∗^*P* < 0.05, compared to NC.*

**TABLE 5:** Effect of S32 on the root growth of plants in ES or RS.

**Soil**	**Experimental soil**			**Reclaimed soil**		
		
**Treatment**	**Root length**	**Root surface area**	**Root volume**	**Root length**	**Root surface area**	**Root volume**
	**(cm)**	**(cm^2^)**	**(cm^3^)**	**(cm)**	**(cm^2^)**	**(cm^3^)**
S32	57.21 ± 3.56^∗^	56.63 ± 9.68^∗∗^	5.99 ± 0.25	33.31 ± 1.65	39.15 ± 5.14^∗^	3.65 ± 0.23^∗∗^
PC	34.52 ± 6.25	28.45 ± 5.59	4.50 ± 0.24	26.85 ± 3.26	24.69 ± 3.63	2.62 ± 0.21
NC	25.81 ± 5.65	25.14 ± 8.50	3.96 ± 0.85	22.32 ± 4.15	23.15 ± 3.00	2.45 ± 0.14

*ES, experimental soil; RS, reclaimed soil; PC, positive control; *B. megaterium As*1.223; NC, negative control. ^∗^*P* < 0.05; ^∗∗^*P* < 0.01 compared to NC.*

**TABLE 6:** Mean leaf and roof P concentrations of plants inoculated with S32 and controls in ES or RS.

**Treatment**	**Leaf P conc.**	**Root P conc.**	**Leaf P conc.**	**Root P conc.**
	**in ES**	**in ES**	**in RS**	**in RS**
	**(μg P/mg)**	**(μg P/mg)**	**(μg P/mg)**	**(μg P/mg)**
S32	200.23 ± 29.13^∗^	45.12 ± 5.67^∗^	123.41 ± 15.51^∗^	36.36 ± 3.02^∗^
PC	143.21 ± 10.46	31.95 ± 3.69	80.24 ± 12.32	28.25 ± 2.56
NC	103.42 ± 11.39	24.19 ± 7.90	69.15 ± 9.54	22.96 ± 1.62

*ES, experimental soil; RS, reclaimed soil; PC, positive control; *B. megaterium As*1.223; NC, negative control. ^∗^*P* < 0.05, compared to NC.*

**TABLE 7:** Effect of S32 on the organic acid release in ES.

**Treatment**	**Oxalic acid (mM)**	**Citric acid (mM)**	**Malic acid (mM)**
S32	93.25 ± 4.02^∗^	52.66 ± 5.88	83.14 ± 14.10^***^
PC	78.14 ± 6.84	51.20 ± 5.08	5.26 ± 3.68
NC	69.00 ± 4.03	25.35 ± 2.31	9.25 ± 2.75

*ES, experimental soil; RS, reclaimed soil; PC, positive control; *B. megaterium As*1.223; NC, negative control. ^∗^*P* < 0.05; ^∗∗∗^*P* < 0.001 compared to NC.*

## Discussion

In the present study, a total of five PSBs were screened from the reclaimed soils. These PSB isolates may possess the potential to be applied in improving soil recovery and crop production. A higher P-solubility capacity of S32 was observed in the Ca_3_(PO_4_)_2_ medium compared to other bacteria and was therefore chosen for further investigation. Its P-solubilization rates for Ca_3_(PO_4_)_2_ and lecithin reached 24.19 and 2.47%, which were nearly quadruple and double that of the positive control *As*1.223, respectively. Furthermore, several insoluble P sources, such as CaHPO_4_, lecithin, powdered PR, AlPO_4_, and FePO_4__,_ could also be dissolved by S32, with P-solubilizing rates of 18.38, 3.07, 0.16, 3.19, and 0.51%, respectively. Additionally, ALP and ACP could also be found in S32, with higher activities (6.94 U/100 mL and 4.12 U/100 mL) than the positive control *As*1.223. Our study also suggested that S32 grew well and was adaptable due to its dependence on multiple carbon sources and tolerance to a broad range of temperatures, pH values, and sodium concentrations. It also indicated that S32 achieved an optimal P-solubilizing rate in the following culture conditions: 5 days incubation, glucose as the carbon source, pH = 7, and temperature = 30°C.

Accumulating reports describe PSBs with the ability to dissolve both Pi and Po. For instance, Li et al. (2013) isolated four Pi-solubilizing bacteria from the plant *Anaphalis lacteal*, and their P-dissolving rates ranged from 65.24 to 315.36 mg/L (D/d ratio of bacteria with the largest P-solubilizing cycle = 1.33). Wei et al. (2014) isolated two P-solubilizing fungi with rates of 1051.69 and 872.18 mg/L on Ca_3_(PO_4_)_2_. Huang et al. (2010) isolated Pi-solubilizing bacteria with a Po-dissolving content of 537.6 mg/L and pH maximal decrease amplitude of 2.79. Lu et al. (2014) screened for Po-solubilizing bacteria with a D/d value of 4.3 and found that the available P in a medium with pH = 7 is 4.8 mg/L. However, the P-dissolving capability of both functions on reclaimed soil has rarely been reported. In the present study, the isolated bacteria S32 displayed higher P-dissolving capacity than the microbes in the above reports. The P-solubilizing cycles for Pi and Po were 4.42 and 5.19, respectively, while the available P in the Pi and Po media were 1256.67 mg/L and 13.05 mg/L, respectively. Remarkably, we tested the P-solubilizing efficiency of S32 in Xiaoyi reclaimed soil, and the data showed that S32 also displayed a considerable P-solubilizing efficiency.

Phosphate solubilizing bacteria mainly belong to *Bacillus* (Babu et al., 2017; Thomas et al., 2018), *Pseudomonas* (Otieno et al., 2015; Paul and Sinha, 2017), *Arthrobacter* (Jiang et al., 2019), *Agrobacterium* (RodrìGuez and Fraga, 1999), *Micrococcus* (Dastager et al., 2010), *Enterobacter* (Jiang et al., 2019), *Vibrio* (Yu et al., 2012), *Serratia* (Behera et al., 2017), *Rhizobium* (Korir et al., 2017), *Aeromonas* (Jha et al., 2012), and *Burkholderia* (Jha et al., 2012). Several fungi display the same function, including *Sclerotium* (Thampi and Bhai, 2017), *Penicillium* (Li et al., 2016), *Aspergillus* (Yin et al., 2017), and *Trichoderma* (Lei and Zhang, 2015). Huang et al. (2010) found that *Pseudomonas* and *Pantoea* are optimal species for their stable P-solubilizing effects. Son et al. (2006) isolated a P-dissolving *Pantoea* from the soybean rhizosphere, and soluble P in its culturing medium reached 900 mg/L. Zhang et al. (2013) isolated another *Pantoea* from the *Caragana microphylla* rhizosphere, and rapidly available P in its Pi culture media reached 4.45 mg/L. Kaur and Reddy (2013) isolated efficient P mineralizing bacteria and tested their efficacy in plant mineral uptake and soil fertility of an organic field. Amongst 12 PSB isolated from an organic field, two isolates were selected for field inoculation based on their RP solubilizing ability, exudation of organic acids, phosphatase and phytase activity and production of indole acetic acid and siderophores. These isolates were identified as Pantoea cypripedii and Pseudomonas plecoglossicida. These isolates significantly increased yield and total P uptake in maize. Soil analysis showed that available P, organic carbon and soil enzyme activities were significantly increased (Kaur and Reddy, 2013). Castagno et al. (2011) isolate 50 PSB strains from a constrained environment such as the Salado River Basin in Argentina. Subsequently, they were found to be related to Pantoea, Erwinia, Pseudomonas, Rhizobium and Enterobacter genera, via 16S rRNA gene sequence analysis. The most efficient isolate, was identified as Pantoea eucalypti, a novel species in terms of plant growth-promoting rhizobacteria (Castagno et al., 2011). Park et al. (2011) isolated 18 different PSB strains from P amended and Lead (Pb) contaminated soils were screened for their efficiency in P solubilization. One PSB was chosen for Pb immobilization and was identified as Pantoea sp. The PSB significantly increased P solubilization by 49.9% in the case of Pantoea sp. for 800 mg/kg of RP addition, respectively, thereby enhancing the immobilization of Pb by 8.25–13.7% (Park et al., 2011). These reports strongly suggested Pantoea sp. a potential candidate for highly efficient PSB. In the present study, the soluble P content in S32 medium reached up to 1256.7 mg/L, demonstrating that S32 possesses a high P-solubilization capacity, even compared to other strains of *Pantoea*.

Over 80% of the P in cropland soil is Pi. Furthermore, Pi components in various types of soil display significant differences, e.g., calcium and magnesium phosphate are dominant in some types of soil, while ferric and aluminum phosphate are more abundant in others (Yu et al., 2012). In the present study, the aluminum phosphate-dissolving rate of S32 was 145.95 mg/L, which was lower than that of the PSB reported by Wang et al. (2013). However, S32 was able to dissolve multiple insoluble P sources other than aluminum phosphate. Different level of soluble phosphates in various forms of P sources affects the solubilization of insoluble phosphates. First, the process of solubilization by the bacteria was documented to be regulated by the external phosphate levels, as also observed for phosphatases (Nahas, 2007). ACP production was reduced while concentration of soluble phosphate was increased (Wang and Lambers, 2019). Second, soil fertilization with P is important to both plant and microorganism growth. Increasing active exudation from roots provides substantial amount of organic acids that enhance solubilization. After soluble phosphate was exhausted, the solubilization process was triggered, with a consequent increase in soluble phosphate in the soil (Nahas, 2007). In the present study, The P-solubilizing efficiency of S32 on different P sources, Ca_3_(PO_4_)_2_, CaHPO_4_, lecithin, PR, AlPO_4_, and FePO_4__,_ was 24.09, 18.38, 2.47, 0.19, 0.51, and 2.62%, respectively.

As heterotrophic bacteria, phosphate solubilizers required carbon source and energy for both the synthesis of new cell material and the oxidation of carbon compounds. Rhizosphere soils present water-soluble C compounds mainly as carbohydrates and organic acids and a small portion and amino acids. It was well known that increasing number of microorganisms was associated with the plant rhizosphere due to its carbon concentration (Nahas, 2007). In the present study, by using glucose, sucrose, starch, fructose, lactose, mannitol, or glycerin as the carbon source, the P solubilization efficiency of S32 was 24.19, 0.75, 0.73, 8.29, 1.30, 7.40, or 5.25%, respectively.

S32 inoculation increased plant height and biomass. In addition to P solubilization activity, PSB was reported to secrete phytohormones that might have an influence on root growth. The extensive root system increased nutrient uptake from the surroundings, which increased plant biomass (Wang and Lambers, 2019). Soluble organic acids could serve as a source of carbon for microorganisms and subsequently affect the rhizosphere microbial environment, as well as plant growth. The plant root development in rice was affected by the application of PSB. Therefore, we also tested the effect of S32 on rice root growth and organic acid production in experimental and reclaimed soil. A plant with S32 inoculation was found to possess fast root growth and release high amounts of organic acids. These results are consistent with the findings of Srivastava et al. (2007) who reported that the addition of PSBs resulted in the release of P and positively affected plant growth. Moreover, Hoffland et al. (1989) found that increased organic acids in the rhizosphere microbial environment were highly efficient at increasing the P-solubility of PSBs. The root development and plant biomass were highly correlated with the higher availability of P; in addition, PSB application may also have some other beneficial effects, such as phytohormone production.

## Conclusion

In conclusion, the bacterium *Pantoea* sp. S32, possessing high dissolving capacity for both Pi and Po, was isolated from alfalfa rhizosphere soil in the reclamation area. S32 showed remarkable P-solubilization rates for different P sources and promoted plant growth, suggesting that the present study provides a potential approach for accelerating minefield recovery and increasing crop output in reclaimed soil.

## Author Contributions

QC performed all the experiments, and collected and analyzed the data. SL conceived the idea, analyzed the data, and wrote the manuscript.

## Conflict of Interest

The authors declare that the research was conducted in the absence of any commercial or financial relationships that could be construed as a potential conflict of interest.
