# Supplementary material for: Identification and Characterization of the Phosphate-Solubilizing Bacterium Pantoea sp. S32 in Reclamation Soil in Shanxi, China
Source: Front Microbiol. 2019 Sep 19;10:2171. doi: 10.3389/fmicb.2019.02171 (PMC6761231; doi:10.3389/fmicb.2019.02171)
Supplement: Supplementary file 1 [file Table_1.docx]

**Supplementary Table 1 Growth experiment of** **the isolated strains**

| Resistant characteristic | Result | | | | | |
| --- | --- | --- | --- | --- | --- | --- |
|  | Y11 | Y14 | Y34 | H22 | S32 |  |
| Growth temperature |  |  |  |  |  |  |
| 4°C | + | - | + | + | + |  |
| 20°C | + | + | + | + | + |  |
| 30°C | + | + | + | + | + |  |
| 37°C | + | + | + | - | + |  |
| 60°C | - | + | - | - | - |  |
| Salt tolerance |  |  |  |  |  |  |
| 2% | + | + | + | + | + |  |
| 5% | - | + | + | + | + |  |
| 7% | - | + | - | - | + |  |
| 10% | - | - | - | - | + |  |
| pH |  |  |  |  |  |  |
| 3 | - | - | - | - | - |  |
| 4 | - | + | - | - | + |  |
| 5 | + | + | + | + | + |  |
| 6 | + | + | + | + | + |  |
| 7 | + | + | + | + | + |  |
| 8 | + | + | + | + | + |  |
| 9 | + | + | + | + | + |  |
| 10 | + | + | + | + | + |  |
| 11 | - | + | - | - | + |  |
| 12 | - | - | - | - | - |  |

+, represents growth; -, represents no growth detected.

**Supplementary Table 2 Cellular fatty acid compositions of the isolated strains and type strains of closely related species**

| Fatty acid | H22 | Y11 | Y14 | Y34 | S32 | FJ168539 | AJ249382 | AJ011507 | AM293566 | JF295057 | DQ504305 | JF295053 |
| --- | --- | --- | --- | --- | --- | --- | --- | --- | --- | --- | --- | --- |
| C12:0 | 7.8 | 1.1 | 3.5 | 8.5 | 5.2 | 2.0 | 1.7 | 3.8 | 3.2 | 4.2 | 4.2 | 4.5 |
| C14:0 | t | － | 10.1 | t | 7.6 | t | t | t | t | 6.9 | 3.0 | 6.7 |
| C16:0 | 24.8 | 14.4 | 32.5 | 22.1 | 27.6 | 27.3 | 30.4 | 29.4 | 35.1 | 26.1 | 30.8 | 27.4 |
| C17:0cyclo | 5.2 | 5.7 | 28.6 | 3.9 | 7.3 | 4.5 | 1.1 | 9.4 | 22.8 | 7.1 | 11.3 | 8.8 |
| C18:1ω7c | 11.7 | 4.0 | 7.0 | 8.6 | 14.6 | 12.9 | 11.6 | 8.5 | 10.2 | 11.8 | 24.4 | 11.0 |
| Summer feature 2 | t | 1.2 | 7.2 | t | 8.0 | 1.6 | t | t | － | 14.3 | 9.0 | 13.9 |
| Summer feature 3 | 37.5 | 14.8 | 4.1 | 38.4 | 25.3 | 27.9 | 35.5 | 30.8 | 18.8 | 26.5 | 10.6 | 23.8 |

-, None detected; t, Trace (< 1%); Summed feature 2, includes iso-C16:1/C14:03-OH and/or C14:03-OH/iso-C16:1; Summed feature 3, includes C16:1ω7c/iso-C15:02-OH and/or iso-C15:02-OH/C16:1ω7c.

**Supplementary Table 3 Results of BIOLOG identification of S32**

|  | Reaction item | Result |  | Reaction item | Result |
| --- | --- | --- | --- | --- | --- |
| A1 | Negative Control | N | E1 | Gelatin | B |
| A2 | Dextrin | MP | E2 | Glycyl-L-Proline | P |
| A3 | D-Maltose | P | E3 | L-Alanine | P |
| A4 | D-Trehalose | B | E4 | L-Arginine | MP |
| A5 | D-Cellobiose | B | E5 | L-Aspartic Acid | P |
| A6 | Gentiobiose | P | E6 | L-Glutamic Acid | P |
| A7 | Sucrose | B | E7 | L-Histidine | P |
| A8 | D-Turanose | B | E8 | L-Pyroglutamic Acid | B |
| A9 | Stachyose | N | E9 | L-Serine | P |
| A10 | Positive Control | P | E10 | Lincomycin | B |
| A11 | pH 6 | P | E11 | Guanidine HCl | B |
| A12 | pH 5 | B | E12 | Niaproof 4 | P |
| B1 | D-Raffinose | B | F1 | Pectin | MN |
| B2 | α-D-Lactose | B | F2 | D-Galacturonic Acid | P |
| B3 | D-Melibiose | B | F3 | L-Galactonic Acid Lactone | P |
| B4 | β-Methyl-D-Glucoside | B | F4 | D-Gluconic Acid | P |
| B5 | D-Salicin | P | F5 | D-Glucuronic Acid | P |
| B6 | N-Acetyl-D-Glucosamine | P | F6 | Glucuronamide | P |
| B7 | N-Acetyl-β-D-Mannosamine | P | F7 | Mucic Acid | P |
| B8 | N-Acetyl-D-Galactosamine | B | F8 | Quinic Acid | P |
| B9 | N-Acetyl Neuraminic Acid | B | F9 | D-Saccharic Acid | P |
| B10 | 1% NaCl | P | F10 | Vancomycin | P |
| B11 | 4% NaCl | B | F11 | Tetrazolium Violet | P |
| B12 | 8% NaCl | N | F12 | Tetrazolium Blue | P |
| C1 | α-D-Glucose | P | G1 | P-Hydroxy-Phenylacetic Acid | N |
| C2 | D-Mannose | P | G2 | Methyl Pyruvate | B |
| C3 | D-Fructose | P | G3 | D-Lactic Acid Methyl Ester | N |
| C4 | D-Galactose | P | G4 | L-lactic Acid | B |
| C5 | 3-Methyl Glucose | P | G5 | Citric Acid | P |
| C6 | D-Fucose | P | G6 | α-Keto-Glutaric Acid | N |
| C7 | L-Fucose | MP | G7 | D-Malic Acid | P |
| C8 | L-Rhamnose | B | G8 | L-Malic Acid | P |
| C9 | Inosine | B | G9 | Bromo-Succinic Acid | P |
| C10 | 1% Sodium Lactate | P | G10 | Nalidixic Acid | N |
| C11 | Fusidic Acid | N | G11 | Lithium Chloride | B |
| C12 | D-Serine | N | G12 | Potassium Tellurite | N |
| D1 | D-Sorbitol | B | H1 | Tween 40 | B |
| D2 | D-Mannitol | P | H2 | γ-Amino-Butyric Acid | B |
| D3 | D-Arabitol | P | H3 | α-Hydroxy-Butyric Acid | N |
| D4 | myo-Inositol | P | H4 | β-Hydroxy-D,L-Butyric Acid | N |
| D5 | Glycerol | P | H5 | α-Keto-Butyric Acid | N |
| D6 | D-Glucose-6-PO_4_ | P | H6 | Acetoacetic Acid | B |
| D7 | D-Fructose-6-PO_4_ | MP | H7 | Propionic Acid | N |
| D8 | D-Aspartic Acid | B | H8 | Acetic Acid | B |
| D9 | D-Serine | L | H9 | Formic Acid | B |
| D10 | Troleandomycin | P | H10 | Aztreonam | B |
| D11 | Rifamycin SV | P | H11 | Sodium Butyrate | N |
| D12 | Minocycline | N | H12 | Sodium Bromate | N |

P: Positive; N: Negative; MP: Mismatched Positive; MN: Mismatched Negative; B: Borderline; and L: Less than the A1 well.
